# Fermentation: improvement of pharmacological effects and applications of botanical drugs

**DOI:** 10.3389/fphar.2024.1430238

**Published:** 2024-08-26

**Authors:** Xinxin Luo, Mosi Dong, Juntong Liu, Naifei Guo, Jing Li, Yan Shi, Yufeng Yang

**Affiliations:** ^1^ Department of First Clinical School, Liaoning University of Traditional Chinese Medicine, Shenyang, China; ^2^ Department of Liaoning Key Laboratory of Chinese Medicine Combining Disease and Syndrome of Diabetes, Liaoning University of Traditional Chinese Medicine, Shenyang, China; ^3^ Department of College of Traditional Chinese Medicine, Liaoning University of Traditional Chinese Medicine, Shenyang, China

**Keywords:** fermentation, botanical drug, reduced toxicity, pharmacological effect, strains abstract

## Abstract

Fermentation is an important concoction technique for botanical drugs. Fermentation transforms and enhances the active ingredients of botanical drugs through specific microbiological processes, ultimately affecting their pharmacological effects. This review explores the use of fermented botanical drugs in areas such as anti-tumor, hypolipidemic, antioxidant, antimicrobial, cosmetology, and intestinal flora regulation. It elucidates the potential pharmacological mechanisms and discusses the benefits of fermentation technology for botanical drugs, including reducing toxic side effects, enhancing drug efficacy, and creating new active ingredients. This article also discussesdelves into the common strains and factors influencing the fermentation process, which are crucial for the successful transformation and enhancement of these drugs. Taken together, this study aimed to provide a reference point for further research and wider applications of botanical drug fermentation technology.

## 1 Introduction

Botanical drugs play a significant role in mainstream medicine because of their clinical effectiveness (Lu et al., 2020). They are abundant in active compounds with medicinal value and have been proven to be effective at treating various diseases, although they may have certain toxic side effects. Fermentation is a biochemical process that usually refers to the conversion of raw materials into desired metabolites by microorganisms through their vital activities. It is a simple, natural, and valuable technology (Ai et al., 2019).

In recent years, the application of fermentation technology in the production of botanical drugs has improved the extraction rate of active ingredients, reduced the dosage of botanical drugs, produced new active ingredients, decomposed toxic ingredients, reduced adverse reactions, enhanced drug efficacy, and broadened the use of botanical drugs in disease treatment. Early fermentation methods relied on natural microorganisms, and the microbial fermentation of botanical drugs, as documented in “The Essentials of the Golden Chamber,” is exemplified by Qu (Yang et al., 2023a). However, these methods have problems, such as impure strains and unclear definitions of medicinal ingredients.

Modern fermentation is an advanced pharmaceutical technology, with the advancement of analytical methods to promote a further understanding of the fermentation strains as well as the composition of botanical drugs, to provide a more stable and safe production process for the fermentation, but also for the elaboration of the mechanism of action of fermented botanical drugs, which breaks through the limitations of natural conditions, the method can select certain strains of bacteria, a clear understanding of the products of fermentation, an in-depth understanding of the mechanism of fermentation, and the pharmacological effects of the drug can be evaluated through compositional analysis and biological assessment, thus improving the efficacy of the drug. The pharmacological effect of a drug can be evaluated by component analysis and biological assessment to improve the efficacy of the drug in a targeted manner, making fermentation an important emerging field with great potential for development. For example, fermentation of *Coix lacryma-jobi* using *Lactobacillus plantarum* NCU137 increases the nutrient content of free amino acids, free fatty acids, soluble dietary fiber, and organic acids; reduces the content of the hazardous substance 2-pentylfuran; improves its safety; and produces a natural preservative, acetic acid, which improves the stability of *C. lacryma-jobi* (Yin et al., 2020). Previous reviews have mostly focused on fermentation technology and its strain application; however, there are few comprehensive analyses of how fermentation enhances the pharmacological efficacy of botanical drugs, the specific advantages of the application of this technology to botanical drugs, and the key factors regulating the fermentation process. This study aimed to fill this gap by systematically reviewing the pharmacological enhancement mechanism of fermented botanical drugs, the advantages of the technology, and the key factors influencing the fermentation process, with the aim of providing a scientific and theoretical basis for further research and wide application of the fermentation technology of botanical drugs.

## 2 Fermentation improves the pharmacological effects of in botanical drugs

**FIGURE 1:**
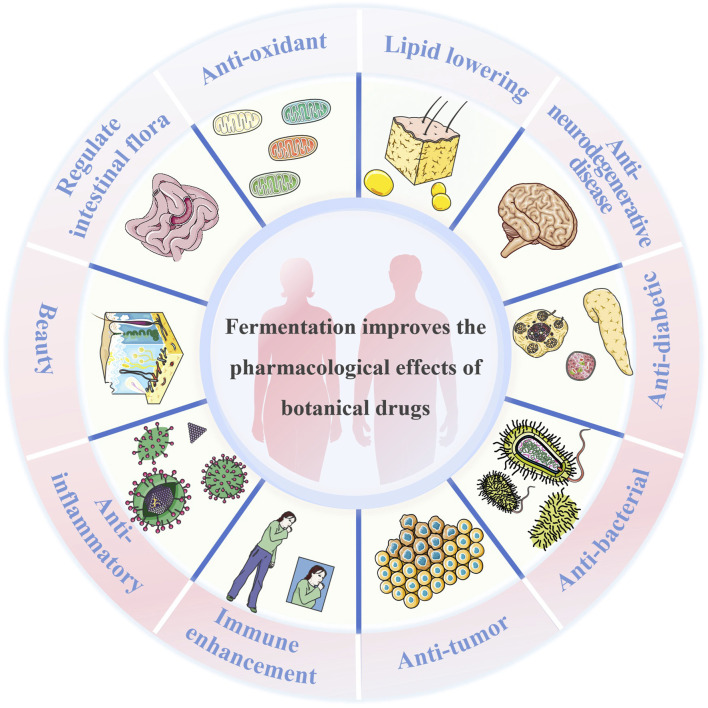
Fermentation improves the pharmacological effects of botanical drugs.

Fermentation of botanical drugs involves a complex series of biochemical reactions and physiological processes. During fermentation, microorganisms utilize polysaccharides, fibers, proteins, and other medicinal components as nutrient sources. This process transforms low-activity ingredients into highly active ones, thereby boosting the efficacy of traditional botanical drugs. Additionally, this leads to the production of new secondary metabolites that play novel roles in the body, as shown in Figure 1.

### 2.1 Metabolic and endocrine regulation

#### 2.1.1 Lipid lowering

Therefore, fermented botanical drugs may exhibit better lipid-lowering effects. Kim et al. (2015) fermented *Panax ginseng* with red koji from *Monascus* spp. to enhance its anti-obesity effects. In female ICR high-fat diet (HFD) -fed rats, fermented *P. ginseng* treatment significantly reduced the diameter of adipocytes per ovary (*P* < 0.01), abdominal fat pads (*P* < 0.05), and abdominal fat thickness. Additionally, fermented *P. ginseng* partially mitigated the HFD-induced weight gain in a dose-dependent manner. Biochemical and histomorphometric analyses confirmed that fermented *P. ginseng* effectively inhibited HFD-induced metabolic disorders, such as hyperglycemia, hyperlipidemia (shown by decreases in serum low-density lipoprotein (LDL), total cholesterol (TC), and triglycerides (TG), and an increase in high-density lipoprotein (HDL), hepatopathy, and nephropathy in a dose-dependent manner. Furthermore, one study indicated that fermented *P. ginseng* has more favorable pharmacological effects on HFD-induced metabolic issues than an equal dose of *P. ginseng* (Kim et al., 2013). This study showed a significant increase in the concentrations of propionic acid, butyric acid, acetic acid, and total short-chain fatty acids (SCFAs) in the colon of type 2 diabetic rats treated with *L. plantarum* plantarum-fermented *Momordica charantia* juice. This increase may be attributed to the regulation of glucose and lipid metabolism, particularly insulin sensitivity and glucose homeostasis, by SCFAs. It also increased the abundance of gut flora compared to untreated diabetic rats. These results suggest that *M. charantia* juice fermented with *L. plantarum* modulates the gut flora and improves glucose and lipid metabolism (Gao et al., 2019). *Momordica charantia* polysaccharide fermented by *L. plantarum* increased the total amount of SCFAs in the colonic contents of a rat model of type 2 diabetes mellitus and significantly improved blood glucose, lipid, insulin levels, and oxidative stress in diabetic rats compared to unfermented *M. charantia* polysaccharide, thus enhancing the anti-diabetic effect of *M. charantia* polysaccharides in rats (Gao et al., 2018). In a separate study, it was found that exposing rats from the HFD + lipopolysaccharide (LPS) group to *L. plantarum*-fermented *Atractylodes macrocephala*, rather than unfermented, led to a significant decrease in the relative weight of abdominal fat, total fat mass, and relative weight of total fat. Additionally, there was a notable reduction in serum TG levels and aspartate transaminase activity, along with a significant increase in serum HDL levels. The study proposed that The anti-adipogenic and anti-obesity effects of fermented *A. macrocephala* may be attributed, in part, to the inhibition of adipogenesis through various mechanisms such as enhancing glucose uptake, modulating the composition of the intestinal microflora to improve gut permeability, and preventing endotoxemia and associated inflammation (Wang et al., 2015). Ji et al. prepared *Rosa roxburghii* Tratt (RRT) juice into temperature-controlled fermentation vials, inoculated with 200 mg/L of Angel active yeast BV818 for 3 months, and obtained fermented RRT juice (FRRT) by sterilization and filtration, which was fed to subgroups of hyperlipidemic rat models. Metabolomic analysis showed that bile acid metabolism in the fermented prickly pear juice-fed group was significantly different from that in the RRT juice-fed group. It was concluded that FRRT helps to reduce the increase in fecal bile acids (e.g., deoxycholic acid and lithocholic acid) induced by a high-fat diet. This was achieved by affecting bacteria, such as *Lactobacillus* and *Staphylococcus*, which in turn inhibited farnesoid X receptor signaling in the liver. This process maintains the balance of bile acids in the enterohepatic circulation and ultimately improves dyslipidemia (Ji et al., 2022). Red yeast rice (RYR) is produced by fermenting ordinary rice using *M. spp*. Recent studies have shown that RYR can prevent weight gain and reduce fat pad weight, while also improving blood lipid parameters, liver enzymes, and leptin levels in HFD-fed rats. These results suggest that RYR has therapeutic potential in the treatment of obesity and hyperlipidemia (Lee et al., 2015).

#### 2.1.2 Anti-diabetic

Accumulating evidence suggests that fermentation has beneficial effects on the anti-diabetic properties of botanical drugs. Fermented *P. ginseng* has been shown to lower fasting blood glucose and HbA1c in a mouse model of type 2 diabetes by increasing serum insulin and lipocalin levels, reducing TNF-α expression, and enhancing the expression of hepatic PPAR-γ and GLUT-2 genes (Jeon et al., 2013). In a separate study involving STZ-treated rats, *P. ginseng* fermented by *Lactobacillus* reduced blood glucose levels during an oral glucose tolerance test. This is accompanied by increased insulin secretion, ultimately resulting in decreased blood glucose levels. A previous study suggested that higher levels of Rb1, Rb2, Rc, Rd, Rg3 (known for their anti-diabetic properties), and ginsenoside Rh2 (which enhances insulin secretion) in fermented *P. ginseng* than in *P. ginseng* may enhance the anti-diabetic effects (Kim H.-J. et al., 2011). Liu et al. fermented *Gynoacemma pentaphllum* using *Lactobacillus* spp. Y5 and found that the content of anthraquinones, polysaccharides, and cyclic allyl glycosides was higher in fermented *Gynoacemma pentaphllum* than in unfermented *Gynoacemma pentaphllum* (*P* < 0.05); the use of fermented *Gynoacemma pentaphllum* in the treatment of diabetic rats for 8 weeks found that the rats showed a decrease in the level of blood glucose and an increase in body weight (*P* < 0.05) (Liu et al., 2023). Yan et al. investigated the hypoglycemic effects of GeGen QinLian Tangand Fermented GeGen QinLian Tang on high-fat diet and Streptozotocin (STZ)-and streptozotocin-induced diabetic rats using a combination of non-targeted metabolomics and targeting analysis. The results of the study showed that fermented GeGen QinLian Tang modulated TC, TG, LDL-C, HDL-C, and fasting insulin levels more than unfermented GeGen QinLian Tang, and fermented GeGen QinLian Tang showed a trend towards better recovery in diabetic rats, suggesting that the fermentation technique enhanced the anti-diabetic properties of GeGen QinLian Tang (Yan et al., 2018). *Lactobacillus plantarum*-fermented DangGui BuXue Tang exhibited improved anti-diabetic effects,,such as inhibiting α-glucosidase, demonstrating antioxidant properties through the 2,2-diphenyl-1-picrylhydrazyl (DPPH) scavenging and T-AOC, and showing anti-glycation capacity in various models (Guo et al., 2020). Li et al. evaluated the hypoglycemic effect of *Lactobacillus shortus* YM 1301-fermented *Polygonatum sibiricum* by analyzing glucolipid metabolism in streptozotocin-induced T2DM rats and a high-fat diet. These findings indicate that fermented *P. sibiricum* demonstrated superior effects on insulin resistance and glycated hemoglobin compared to unfermented *P. sibiricum*. Furthermore, fermented *P. sibiricum* not only boosts AMPK activation, but also increases the ratio of phosphorylated AKT/AKT to mitigate issues with glucose tolerance and insulin resistance (Li C. et al., 2021). Yang et al. investigated the effects of solid-state fermentation products of *Astragalus membranaceus* and *Paecilomyces cicadidae* in diabetic nephropathy (DN) and found that fermented *A. membranaceus* had a significant mitigating effect on mice with diabetic nephropathy (DN), and that fermented *A. membranaceus* significantly reduced urinary proteins, serum creatinine, and blood urea nitrogen in mice with DN. Compared to unfermented *A. membranaceus*, fermented *A. membranaceus* had a better effect on improving renal structure in DN. The results of the *in vitro* experiments indicated that fermented *A. membranaceus* enhanced the autophagy of podocytes, which may delay the onset of DN by inhibiting the PI3K/AKT/mTOR signaling pathway (Yang et al., 2020).

### 2.2 Immunity and anti-infection

#### 2.2.1 Immune enhancement

Clinical and experimental studies have shown that fermented botanical drugs can modulate immunity through various pathways. Wang et al. fermented *Platycodon grandiflorum* using *Lactobacillus rhamnosus* 217-1 and found a significant increase in polyphenol and flavonoid content in the fermented group compared to the unfermented *P. grandiflorum* (*P* < 0.05), and a significant difference in the scavenging of DPPH free radicals in the fermented group compared with the control group (*P* < 0.01). Treatment of the UC mouse model revealed that fermented *P. grandiflorum* liquid significantly restored dextran sulfate sodium-induced colonic shortening (*P* < 0.01) and significantly increased the mRNA expression of OCLN and TJP1 (*P* < 0.01, *P* < 0.05 vs. Model), suggesting that fermented *P. grandiflorum* may have improved autoimmunity in mice (Wang et al., 2022b). Li et al. examined the impact of fermentation broth (GLFB) on dexamethasone (DEX)-induced immunosuppression in rats using *Lactobacillus acidophilus* and *Bifidobacterium bifidum* for *in vitro* fermentation of aqueous extracts from *Ganoderma lucidum* substrates. These findings indicate that the extract formed through probiotic fermentation modified the composition of the main ganoderic acid components. Furthermore, GLFB notably enhanced immunity and intestinal integrity and rectified intestinal flora dysbiosis in DEX-treated rats (Li Y. et al., 2021). Sun et al. compared the immunomodulatory activity of unfermented Yupingfeng polysaccharides and trans-oligosporic rhizobacteria in weaning Rex rabbits and found that fermented Yupingfeng polysaccharides significantly increased intestinal *Lactobacillus* and Bifidobacterium populations, while reducing the presence of opportunistic pathogens, such as Enterobacteriaceae bacteria and *Streptococcus*. This effect may be attributed to the fact that the fermentation products increased the number of probiotic bacteria involved in colonization antagonism, competition for nutrients, and ultimately enhanced biobarrier function. Thus, inhibition of harmful bacterial proliferation and enhancement of intestinal immunity were effectively achieved (Sun et al., 2016)

#### 2.2.2 Anti-inflammatory

Inflammation is the adaptive response of an organism to infection and tissue damage that restores homeostasis. A successful acute inflammatory response involves the elimination of infectious agents and dissolution and repair. There is emerging evidence that the fermentation of botanical drugs can enhance their anti-inflammatory effects. Yong et al. found that fermentation of *Curcuma longa* by *Lactobacillus* fermentum significantly increased curcumin content by 9.76% and effectively decreased the expression of pro-apoptotic tumor necrosis factor-α and Toll-like receptor-4 in RAW 246.7 cells compared with unfermented *C. longa*. Western blotting analysis further showed that the anti-inflammatory activity of fermented *C. longa* was achieved by inhibiting the c-Jun N-terminal kinase (JNK) signaling pathway, whereas unfermented *C. longa* was not. (JNK) signaling pathway, whereas unfermented *C. longa* did not (Yong et al., 2019). Similarly, another study showed that *A. membranaceus* fermented by *L. plantarum* enhanced its anti-inflammatory properties. Fermentation of *Astragalus membranaceus*resulted in inhibition of lipopolysaccharide (LPS)-induced NO production and downregulation of TNF-α, iNOS, COX-2 and nuclear factor-κB (NF-κB) expression in RAW 264.7 cells (Park et al., 2021). The anti-inflammatory effects of *P. ginseng* fermented with *Lacto. plantarum* KP-4 was more effective than untreated *P. ginseng*. LC–MS/MS analysis showed that the major ginsenosides decreased and the minor ginsenosides Rg3, F2, and Rh1 increased significantly in *P. ginseng* after fermentation, whereas new minor ginsenoside compounds, CK and Rh3, were generated, suggesting that the anti-inflammatory effect of fermented *P. ginseng* was closely related to changes in ginsenosides (Fan et al., 2021). Compared with unfermented *Paeonia lactiflora* extracts, *P. lactiflora* extracts fermented by *L. shortus* 174A significantly increased total phenolic content, decreased intracellular ROS levels, and inhibited NO release, as well as reduced gene expression of the inflammatory cytokines IL-6, TNF-α, and IL-1 (Shakya et al., 2021). Liu et al. used a mouse model of dextran sodium sulfate-induced ulcerative colitis to study the anti-ulcerative effects of fermented and unfermented *Lycium barbarum* juice. The results of this study showed that *L. plantarum*, *Lactobacillus royale* and *S . Streptococcus* spp. Both fermented and unfermented *L. barbarum* juices exhibited positive anti-inflammatory effects. In addition, fermented *L. barbarum* juice exhibited more significant modulatory effects on serum and colonic inflammatory cytokines and related enzymes, including T-SOD, NO, IL-1β, IL-4 and IL-10, compared to unfermented *L. barbarum* juice (Liu et al., 2021). Oh et al. investigated the effects of a *Morus alba* extract fermented by *L. acidophilus* A4 on intestinal mucositis in a rat model induced by 5-fluorouracil. Results demonstrated that these interventions led to improvements in inflammation by upregulating MUC2 and MUC5AC gene expression, enhancing mucin production, and reducing IL-1β expression and myeloperoxidase levels. Notably, fermented *M. alba* extract exhibited the most significant protective effect compared to the control group (Oh et al., 2017). The production of endotoxins results in increased intestinal permeability, allowing toxins and bacteria to breach the mucosal barrier, triggering the release of inflammatory mediators and causing further harm to the intestine (Di Michele, 2022). Bose et al. evaluated the *in vitro* and *in vivo* protective effects of fermented and unfermented *Coptis chinensis* on LPS-induced rats. The results of the study showed that endotoxin levels were significantly reduced in mice treated with fermented *C. chinensis* compared to the unfermented group (*P* < 0.05), whereas the combination of fermented *C. chinensis* extract and probiotics showed stronger anti-inflammatory activity *in vitro* and was more effective in reducing LPS-induced intestinal permeability (Bose et al., 2012).

#### 2.2.3 Anti-bacterial

Pathogenic bacteria, particularly those related to foodborne illnesses, pose a significant threat to human health owing to their secretion of harmful toxins. The World Health Organization reports that approximately 1.55 billion cases of diarrhea occur worldwide annually, leading to three million deaths in children under 5 years of age, with approximately 70% of these cases attributed to foodborne pathogens (Ju et al., 2019). Studies have shown that Fermented botanical drugs exhibit strong antibacterial properties (Kanklai et al., 2020). A previous study demonstrated that the fermentation of *Portulaca oleracea* exhibited significant antibacterial activity against *Campylobacter* jejuni, a major microorganism that causes diarrheal disease. Among the various strains, *Leuconostoc mesenteroides* KACC 12312 and *lactic acid bacteria* isolated from *P. oleracea* show the highest activity (Bae, 2012). Another study investigated the antibacterial activity of fermented *Hippophae rhamnoide* juice against 10 foodborne pathogens and found that its effectiveness increased after fermentation with *L. plantarum* RM1. An increase in the phenolic content and acidity during fermentation appears to enhance the antimicrobial potential of fermented *H. rhamnoide* juice (El-Sohaimy et al., 2022). *Glycine max* fermented with Rhizopus oligosporus exhibit significant antibacterial activity against Rhizopus oligosporus aureus and *Bacillus subtilis*. This effect may be linked to the synthesis of long-chain polyunsaturated acids and fatty acid antibacterial compounds during fermentation process (Kusumah et al., 2020). *Magnolia officinalis* extracts fermented with Aspergillus niger exhibited enhanced antibacterial activity against a variety of tested strains (*Escherichia coli*, *Staphylococcus aureus*, *Bacillus subtilis*, *Staphylococcus* epidermidis, Propionibacterium acnes, Epidermophyton floccosum, and methicillin-resistant *S. aureus*), with a significant 8-to 20-fold increase compared with unfermented extracts (Wu et al., 2018a).

### 2.3 Digestive system and microecology

#### 2.3.1 Regulate intestinal flora

Recent studies have demonstrated that fermented botanical drugs can influence the intestine and play a significant role in regulating intestinal flora. Zhao C. et al.(2021) found that *Monascus*-fermented *P. ginseng* could reverse the decrease in species abundance and diversity of the intestinal flora in rats fed a high-fat diet. It has been theorized that this process may elevate the relative abundance of Prevotella while decreasing the relative abundance of Muri, thus enhancing the hydrolysis of ginsenosides and regulating cholesterol levels to improve lipid metabolism disorders. In a murine model of alcoholic liver injury, *P. ginseng* fermented with *Lactobacillus fermentum* was found to enhance the growth of beneficial probiotics such as *Lactobacillus* and *Bifidobacterium*, leading to significant improvements in alcohol-induced intestinal permeability and short-chain fatty acid (SCFA) levels. The study also demonstrated that fermented *P. ginseng* positively affected other SCFA-producing bacterial strains, such as Allobacterium, Ruminococcus, and *Streptococcus*, while reducing the abundance of bacteria linked to inflammatory conditions, ultimately ameliorating intestinal disorders and alleviating inflammation (Fan et al., 2019). Total flavonoids, total triterpenes, and related short-chain fatty acids were significantly higher in *H. rhamnoide* fermentation liquid. The study indicated that *H. rhamnoide* fermentation liquid effectively improved alcohol-induced liver injury.By measuring gut microbiota in mice feces samples, we found that the high-dose group of SFL reversed the declining trend of the gut microbiota Firmicutes/Bacteroidetes (F/B) ratio caused by alcohol, reducing the number of gram-negative bacteroidetes. These findings suggest that SFL has the potential to prevent alcoholic liver disease and modulate intestinal flora composition (Ran et al., 2021). Zhang et al. examined the metabolic functions of the gut microbiota using PICRUSt analysis of 16S rRNA gene sequences and found that *Laminaria japonica* fermented by *Lactobacillus shortcombicus* FZU0713 affected primary and secondary bile acid biosynthesis in a rat model of hyperlipidaemia. It also significantly affected the expression of certain mRNAs associated with lipid metabolism and bile acid homeostasis, including BSEP, CYP7A1, LDLR, HMGCR, CD36, and SREBP1-C. These findings suggest that the microbial fermentation of *L. japonica* has the potential to modulate body health and ameliorate metabolic disorders by regulating the gut flora (Zhang et al., 2021). Duan et al. (2024) found that *L. barbarum* juice fermented with *Lactobacillus paracasei* E10, *L. plantarum* M, and *L. rhamnosus* LGG enhanced intestinal integrity, remodelled the intestinal microbiota by increasing the presence of *Bacteroides* and *Lactobacillus* spp., and altered intestinal microbial metabolites, such as Kyotoferrin, indolactic acid, and N-methyl-serotonin, compared to unfermented *L. barbarum* juice. Correlation analyses showed that E10F, MF, and LGGF increased *Lactobacillus*, indolactic acid, and N-methylserotonin levels, which were positively correlated with reduced inflammation and enhanced hepatic and intestinal functions. This study used ultra-performance liquid chromatography-Q Exactive Hybrid Quadrupole-Orbitrap Mass Spectrometry (UPLC-Q-Exactive-MS) to identify differential metabolites in *A. membranaceus* fermented by *L. plantarum*, and the results showed that 11 different metabolites, such as raffinose, progesterone, and uridine, might contribute to the fermented *A. membranaceus* ability to alleviate colitis. In addition, fermented *A. membranaceus* alters the structure of the gut microbiota and enriches Akkermansia and Alistipes, which are positively correlated with short-chain fatty acid production. Compared to mice treated with unfermented *A. membranaceus*, mice treated with fermented *A. membranaceus* showed more pronounced expression of intestinal tight junction and mucus secretory proteins ZO-1, occludin, and MUC2, as well as modulation of apoptosis of intestinal epithelial cells (IECs), which verified the reparative effect of fermented *A. membranaceus* on the intestinal mucosal barrier. Mice supplemented with fermented *A. membranaceus* showed a more pronounced expression of intestinal tight junction proteins and mucus secretory proteins ZO-1, occludin, and MUC2, while apoptosis of intestinal epithelial cells (IECs) was also regulated, which verified the reparative effect of fermented *A. membranaceus* on the intestinal mucosal barrier (Li et al., 2022). Lee et al.(2017) incorporated *Lactobacillus bulgaricus*, *Streptococcus thermophilus*, and *L. acidophilus* into the fermentation process of *M. alba* extracts. These findings indicate that fermented *M. alba* extract leads to a decrease in intestinal transit time and fecal particles in the colon, while also increasing the presence of *lactobacilli* in the stool, thus potentially aiding in the prevention of constipation. Guo et al. (2022) found that a Sijunzi decoction fermented by *L. plantarum* significantly increased the expression levels of AQP1, ZO-1, and occludin mRNA in aquaporin (AQP), and enhanced the level of AQP4 mRNA expression in aquaporin (AQP) deficiency induced by sodium sporatricepsin. This restoration of the intestinal epithelium structure and function, repair of membrane permeability and intestinal barrier, and regulation of water inlet and outlet ability effectively treated diarrhea in rats.

### 2.4 Neuroprotection and anti-tumour therapy

#### 2.4.1 Anti-neurodegenerative disease

Recent studies have shown that fermented botanical drugs exert neuroprotective effects. Kim et al. investigated the potential anti-neurodegenerative properties of yeast-fermented *Ziziphus jujuba* using a rat disease rat induced by amyloid beta25–35. These results demonstrate that fermented *Z. jujuba* has a positive impact on cognitive function and memory by reducing amyloid beta25–35-induced oxidative stress. Notably, fermented *Z. jujuba* exhibited stronger antioxidant effects on malondialdehyde and nitric oxide in tissues and serum than unfermented *Z. jujuba*, which could be attributed to the enhanced presence of bioactive compounds resulting from fermentation (Kim et al., 2021). Fermentation of *P. ginseng* with *L. paracasei* has been demonstrated to improve spatial memory deficits caused by cerebral ischemia and β-amyloid injection, as well as protect hippocampal neurons from apoptotic death in rats (Nagao et al., 2019). Mei et al. found that Shuan-Tong-Ling fermented by *Lactobacillus*, *Bacillus acetate*, and *Saccharomyces* inhibited neuronal inflammation and apoptosis through the activation of the SIRT1 signaling pathway, which is beneficial for the treatment of cerebral ischemia/reperfusion injury (Mei et al., 2017). A recent study found that fermented *Zingiber officinale* extracts exhibited greater neuroprotective properties than unfermented *Z. officinale* extracts. This was attributed to the metabolic transformation of *Z. officinale* extracts by Aspergillus niger, resulting in the conversion of side chain α,β-unsaturated ketones to 6-paradol and other related metabolites. The increased bioavailability of 6-paradol was identified as a key factor in the enhanced neuroprotective effects (Park et al., 2016).

#### 2.4.2 Anti-tumor

With the increasing research on fermented botanical drugs, it has been found that Botanical components treated with specific microorganisms have also shown significant effects in anti-tumor. Yim et al. found that fermentation of So-Cheong-Ryong-Tang (CY) by *L. fermentum* altered the composition of CY and enhanced the anticancer properties of the fermented CY preparation (FCY). In a xenograft assay, FCY significantly inhibited the tumor growth of subcutaneously injected cancer cells compared to unfermented CY. These findings suggest that the anticancer efficacy of FCY can be enhanced by modifying its active ingredients via *Lactobacillus* fermentation (Yim et al., 2015). Melanoma is one of the most serious malignant epidermal cancers worldwide. *Glycine max* freeze-dried extract and *G. max* water extract were obtained from soybeans fermented with Natto and *B. subtilis* and were evaluated as potential antimelanoma agents. Cytotoxicity experiments demonstrated that *G. max* freeze-dried extrac and *G. max* water extract exhibit notable antimelanoma properties by inhibiting the AMPK signaling pathway, thereby inducing oxidative stress in cancer cells and ultimately triggering apoptosis (Chou et al., 2021).

### 2.5 Anti-aging and beauty

#### 2.5.1 Anti-oxidant

Research shows that Microbial fermentation plays a crucial role in enhancing the antioxidant capacities of botanical drugs. Furthermore, fermentation can boost the antioxidant activity of plant extracts by increasing the levels of phytochemicals, particularly polyphenols, antioxidant polysaccharides, and antioxidant peptides generated through microbial hydrolysis or biotransformation (Zhao Y.-S. et al., 2021). Li et al. fermented *L. barbarum* juice using food-derived bacterial strains, including *B. subtilis*, *Bacillus licheniformis*, *Lactobacillus reuteri*, and a mixed strain of *L. rhamnosus* and *L. plantarum*. The results showed that the fermentation of *L. barbarum* juice affected the conversion of free and bound forms of phenolic acids and flavonoids and increased their antioxidant capacity (Liu et al., 2019). Another study demonstrated that fermenting pumpkin juice with five species of *Lactobacillus spp*. resulted in abundant organic acids from *Lactobacillus casei paracasei*, whereas *L. plantarum*, *L. acidophilus*, and *Lactobacillus swissii* exhibited strong DPPH and hydroxyl radical scavenging abilities. These abilities are positively correlated with the content of vanillic acid and erucic acid (Sun et al., 2022). Khan et al. *Dimocarpus longan* underwent fermentation with selected strains of lactic acid bacteria (*L. plantarum subsp. Plantarum and L. mesenteroides*). The fermentation process was shown to increase the ferric reducing antioxidant power. Furthermore, fermentation leads to a decrease in the levels of free amino acids responsible for the bitter taste, while increasing the presence of amino acids with antioxidant properties (Khan et al., 2018). It was found that *H. rhamnoide* juice fermented by *L. plantarum* increased 2,2-diphenyl-1-picrylhydrazyl (DPPH) and 2,2’azobis-(3-ethylbenzothiazoline-6-sulfonic acid) (ABTS) scavenging activity, which was positively correlated with the increase in phenolic compounds (El-Sohaimy et al., 2022). This aligns with the results of Tkacz et al. (2020), who showed that fermentation leads to the biotransformation of flavonols in *H. rhamnoide* juice, resulting in enhanced antioxidant activity (Tkacz et al., 2020). In addition, studies have indicated that *L. spp*. enhance the antioxidant properties of fermented *Perilla frutescens* seeds. During fermentation, *lactic acid bacteria* release antioxidant phenolics from the cellular matrix of *P. frutescens* seeds through secondary metabolic pathways or extracellular enzymatic action. These phenolics can neutralize free radicals either through hydrogen atoms or direct electron transfer (Kawee-Ai and Seesuriyachan, 2019). Fang et al. examined the impact of *C. lacryma-jobi* fermented by *L. reuteri* on H2O2-induced oxidative stress. The results showed that the fermented group exhibited significant activation of the PI3K/AKT signaling pathway, reduction in intracellular ROS levels, upregulation of the COL-I gene, and a notable decrease in MMP-1 expression, indicating an antioxidant effect (Fang et al., 2022). Furthermore, *A. membranaceus* fermented by *Lactobacillus* MG5125, *B. bifidum* MG731, and *Lactobacillus* MG741 reduced hepatic aspartate aminotransferase, alanine aminotransferase, and lipid peroxidation in the tBHP-injected mouse model. The fermentation of *A. membranaceus* leads to enhanced isoflavone sapogenins through glycoside hydrolysis, thereby boosting the antioxidant activity of *A. membranaceus* (Lee and Kang, 2022).

#### 2.5.2 Beauty

Cosmetics and pharmaceutical industries are seeking new products or enhancements to existing products that contain innovative active ingredients. Botanical products and their active constituents remain consistently popular with consumers, with fermented botanical drug products being particularly popular in Asian countries and gaining more attention in global markets. Lee et al. (2022) mixed root extracts of *Taraxacum mogolicum*, *Arctium lappa*, *Anemarrhena asphodeloides*, *Pueraria lobata*, and *Nelumbo nucifera* fermented with *Saccharomyces cerevisiae* to promote the proliferation and migration of human keratinocytes (HEKa) and fibroblasts (HDF), exhibiting anti-aging properties and potential as active cosmetic ingredients. Fermented *Angelica sinensis* has been shown to enhance extracellular matrix damage induced by UVB irradiation by increasing the production and release of procollagen type-1, while reducing the expression of MMP-1 and elastase in HaCaT (human keratinocyte) or Hs68 (human foreskin fibroblast) skin cells (Kim et al., 2010). Ho et al. performed *in vitro* and *in vivo* experiments on FB-ChiBai (consisting of extracts of *A. macrocephala*, *P. lactiflora*, *Bletilla striata*, *Poria cocos*, *Dictamnus dasycarpus*, *Ampelopsis japonica* and *Tribulus terrestris*), a mixture fermented by *L. rhamnosus*. It was found that FB-ChiBai inhibited melanogenesis in α-melanocyte-stimulating hormone-induced B16F0 mouse melanoma cells without cytotoxicity in the *in vitro* assay. *In vivo*, FB-ChiBai inhibits melanogenesis by inhibiting the CREB/MITF/tyrosinase signaling pathway. These results suggested that FB-ChiBai has the potential to protect against UV-B radiation (Ho et al., 2021). The anti-tyrosinase and anti-wrinkle activities of *Citrus aurantium* flowers fermented by *Lactobacillus brevis* were found to be 5.2-fold and 4.29-fold higher, respectively, than those of the unfermented group. Furthermore, the fermented extract of *L. brevis* exhibits significant inhibitory activity against wrinkle-related enzymes activities (Chen et al., 2022a). Cui et al. (2022) found that fermented *L. japonica* offers skin-protective benefits. The use of *Bacillus siamensis* in the fermentation process of *L. japonica* has been shown to improve the release of its bioactive compounds and exhibit positive effects such as tyrosinase inhibition, skin repair, and anti-wrinkle properties. In a separate study, *B. subtilis* natto-fermented R. *astragali* was found to have skincare benefits. This fermented product notably increased hyaluronic acid production in primary human epidermal keratinocytes and human dermal fibroblasts. It also increased the expression of hyaluronate synthase 3 and hyaluronate synthase 2 mRNA in HaCaT cells and human fibroblasts compared to that in the non-fermented group. These positive effects on the skin can be attributed to isoflavone glycosides or other metabolites formed from the major isoflavones during fermentation (Hsu and Chiang, 2009). A recent study demonstrated that fermentation by *L. brevis* enhanced the anti-wrinkle and whitening effects of *P. ginseng* and reduced its toxicological potential. Higher concentrations of uronic acid, polyphenols, flavonoids, and antioxidant properties were observed in fermented *P. ginseng* compared to non-fermented *P. ginseng*. In addition, fermented *P. ginseng* exhibited stronger tyrosinase and elastase inhibitory effects than *P. ginseng*. Furthermore, fermentation increases the levels of ginsenoside metabolites including Rg3, Rg5, Rk1, compound K, Rh1, F2, and Rg2 (Lee et al., 2012). Cha et al. demonstrated that inhibiting tyrosinase activity and melanin synthesis using a 0.3% (w/v) optimal dose of fermented *Aloe barbadensis* extract was more effective than arbutin and aloesin, common commercial skin-lightening ingredients. Furthermore, fermented *A. barbadensis* extract significantly reduced the expression of microphthalmia-associated transcription factor (MITF), tyrosinase-related protein-1 (TYRP-1), TYRP-2, and tyrosinase (TYR) genes, suggesting a mechanism for inhibiting melanogenesis in the MITF/TYRP-1/TYRP2/TYR pathway. Additionally, a combination of fermented *Glycyrrhiza glabra*, *Broussonetia papyrifera*, *A. sinensis*, *A. macrocephala*, *P. cocos*, *M. alba*, and *Paeonia labiflora* (2% each) with Phellinus linteus exhibited antimelanogenic activity against B16F0 mouse melanoma cells tested in culture (Cha et al., 2012). The study used *L. plantarum*, *L. rhamnosus*, *L. casei*, *Lactobacillus gattii*, and other *Lactobacillus* strains to ferment the *Lepidium meyenii* extract, and found that the secretion of the inflammatory mediator nitric oxide was considerably lower in the fermented than in the unfermented extracts in the RAW264.7 cells, suggesting an effective anti-inflammatory action of the fermented *L. meyenii* and the inhibition of tyrosinase activity, melanin synthesis and melanogenesis (Yang et al., 2023a).

## 3 Application advantages of fermented botanical drugs

**FIGURE 2:**
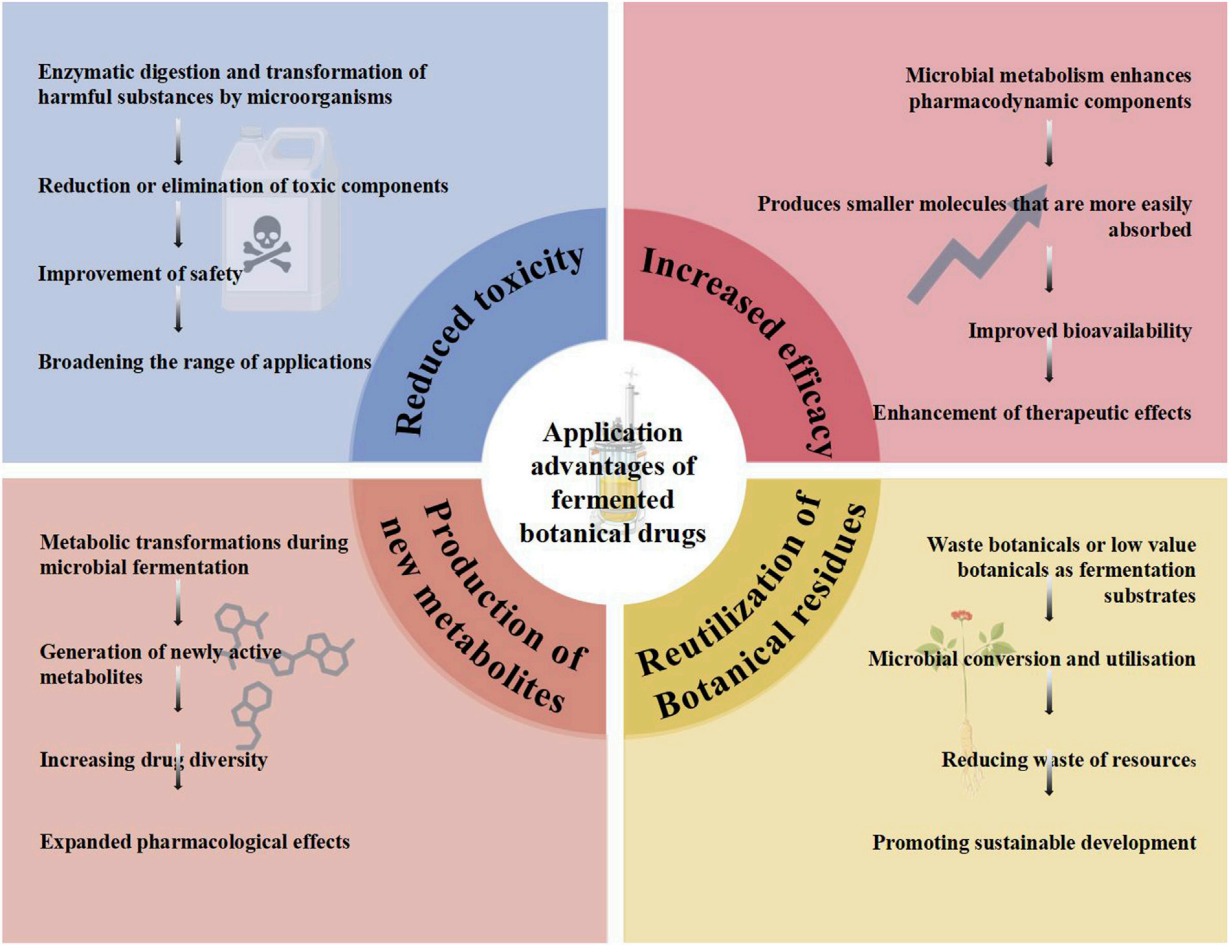
Application advantages of fermented botanical drugs.

Fermentation has become one of the most important methods for processing botanical drugs (Li et al., 2020). Fermentation improves the solubility and bioavailability of botanical drugs, reduces their toxicity of botanical drugs, and improves their safety and efficacy of botanical drugs. The interaction between microorganisms and botanical drugs in the fermentation process can also produce new active ingredients, and the fermentation method applied to dregs of botanical drugs can also realize the multi-level utilization of traditional botanical drug resources. The advantages of applying fermentation technology to traditional botanical drugs are shown in Figure 2.

### 3.1 Reduced toxicity

Several studies have found that fermentation alters or breaks down the toxic components of botanical drugs and reduces their cytotoxicity compared to non-fermented botanical drugs. Botanical drugs often contain toxic macromolecular substances. In addition, when they enter an organism, they produce irritants that can cause toxicity or other side effects. Fermentation can reduce the adverse effects and toxicity of botanical drugs containing cytotoxic compounds such as heavy metals, toxic glycosides, and toxic proteins (Li J. et al., 2021; Hussain et al., 2016; Li et al., 2022). Aconitine is a potential preventive and therapeutic agent for various diseases; however, it carries the risk of ventricular tachyarrhythmias and cardiac arrest, which can be fatal (Zhou et al., 2021). Therefore, fermentation with probiotics can effectively alleviate this problem. Aconitine is the main ingredient in Huafeng Dan Yaomu and is used to treat hemiplegia, epilepsy, and facial paralysis. The production of Huafeng Dan Yaomu involves fermentation. Cao et al. (2020) investigated the changes in toxic alkaloids during the fermentation process using high performance liquid chromatography (HPLC) and analyzed the changes in the microbial community during the fermentation process using the Illumina MiSeq platform and found that the contents of the toxic alkaloids aconite, neoaconitine, and hypoaconitine decreased during the fermentation process; at the same time, the lower toxicity of benzoylneaconitine and benzoyl hypaconitine, which are less toxic. *Aristolochia debilis*, a traditional Chinese herb used for blood pressure reduction and pain relief, is known for its anti-diarrheic properties. However, due to the nephropathy associated with *aristolochic* acids, botanical drugs have been prohibited for clinical use in China by the State Food and Drug Administration (Yang et al., 2012). Nevertheless, a study on fermenting *A. debilis* with six different medicinal fungi and analyzing the products of aristolochic acids using HPLC-ESI-TOF-MS demonstrated a significant decrease in the content of aristolochic acids after fermentation (Liu et al., 2018). Guo et al. found that ginkgolic acid was significantly reduced to safe levels using *Ginkgo biloba* fermented with *Bacillus subtilis* natto (No. 1A752). The study suggested that the reduction in ginkgolic acid may be due to enzymes secreted by the probiotic bacteria degrading ginkgolic acid or metabolites produced by the probiotic bacteria (e.g., proteins or amino acids), altering the structure of ginkgolic acid (Guo et al., 2018). The removal of toxic ingredients from botanical drugs may lead to a loss of efficacy or even survival due to toxicity, ultimately failing to achieve the intended therapeutic outcome. Fermentation plays a crucial role in efficiently transforming the toxic components of these medicines, generating new chemical compounds that are highly effective and have low toxicity. This process enables the dosage of toxic botanical drugs to be controlled within safe limits, without compromising their therapeutic benefits. Such fermentation-based approach distinguishes itself from traditional preparation methods and forms the theoretical foundation for the concept of 'removing the poison and preserving the effect’ in toxic botanical drugs.

### 3.2 Increased efficacy

Most active ingredients of botanical drugs are contained within the cell walls. These dense and hard cell walls act as barriers, preventing the active ingredients from easily leaching out and being absorbed by the body. Microorganisms play a crucial role in the extraction of active ingredients. They target different cell wall components, secrete various extracellular enzymes, and break down the tight structure of the cell. This process enlarges the gaps between the cells, allowing for better diffusion of substances in and out of the cells. Ultimately, this not only enhances the extraction rate of active ingredients but also improves their absorption and utilization. Traditional Chinese botanical drugs are usually administered orally, which results in low bioavailability of the active ingredients. However, during microbial fermentation, extracellular enzymes such as cellulase and pectinase produced by microorganisms enter the culture medium, causing the botanical cells to rupture and exposing the active ingredients. Additionally, fermentation is known to improve the absorption and bioavailability of botanical extracts by aiding in the production or conversion of active components into metabolites, or by producing low-molecular-weight substances such as aglycones from glycosides (Joo et al., 2009). Previous studies have shown that microorganism-mediated fermentation of botanical drugs can synthesize important microbial and vegetative secondary metabolites that degrade macromolecular organic substances into small active compounds and increase their therapeutic effects (Stanton et al., 2005; Hussain et al., 2016; Su et al., 2022). Studies have shown that fermented medicinal botanical drugs have stronger anti-ovarian cancer, antioxidant, and neuroprotective effects than unfermented botanical drugs.(Huang et al., 2017; Fu et al., 2014; Tan et al., 2016). Oxalic acid has physiological benefits for human health; however, high concentrations of oxalic acid, an anti-nutritional factor, can bind to essential minerals in the body to form insoluble oxalates, leading to limited mineral absorption and hyperoxaluria (Gomathi et al., 2014; Shi et al., 2018). Filannino et al. used different 8 strains of *Lactobacillus* isolated from fruits, vegetables, and the honeybee intestinal tract as single fermenters, and each strain was selected based on specific technical and functional traits. The determination of organic acids by high performance liquid chromatography (HPLC) revealed that the oxalic acid content in *P. oleracea* fermented by *Lactobacillus kunkeii B7* and *L. plantarum POM1* was reduced (approximately 30%), which promoted the absorption of nutrients in *P. oleracea* (Filannino et al., 2017). Recent research has shown a direct correlation between the oral administration of probiotics and reduced urinary oxalate excretion in both rats and humans. This indicates that probiotic fermentation could be a promising approach to reduce the antinutritional factors of botanical drugs, making them more suitable for human consumption (Abratt and Reid, 2010; Mehra et al., 2022). Nan et al. explored the biotransformation of ginsenosides by *L. fermentum* KP-3 in a high-fat diet-fed mouse model. The results showed that The ginsenoside content increased from 0.746 mg g-1 to 0.939 mg g-1 after fermentation, and the fermentation of *P. ginseng* by *L. fermentum* KP-3 significantly reduced serum TC and LDL levels and inhibited the sharp increase in hepatic alanine aminotransferase (ALT) and alanine oxaloacetate aminotransferase (AST) levels compared to the unfermented group. The significant decrease in the ginsenoside Rb1 content during fermentation was attributed to the conversion of Rb1 to the minor ginsenosides Rg3 and F2 via fermentation, as confirmed by TLC analysis. Ginsenoside Rg3 can be produced directly by eliminating two glucose units at the C-20 position of ginsenoside Rb1, whereas ginsenoside F2 can be derived from ginsenoside Rb1 by removing one glucose unit at the C-3 and C-20 positions of ginsenoside Rb1 (Nan et al., 2018). Major ginsenosides can be converted into deglycosylated and minor ginsenosides, with the general belief that these converted ginsenosides exhibit enhanced bioavailability and more potent pharmacological activity (Bae et al., 2011; Kim S. H. et al., 2011). Solid-state fermentation of *Salvia miltiorrhiza*, *P. grandiflorum*, *Huperzia serrata*, and *G. glabra* using *Aspergillus oryzae* NCH 42, an extracellular ellagitannase-producing fungus, for 5 days at 30°C revealed that extraction of phenolic substances from all four phytomedicines was significantly increased by fermentation, and that the antioxidant and bacteriostatic activities of the extracts were enhanced (Wen et al., 2013). During fermentation of botanical drugs, specific glycosides are converted into smaller hydrophobic molecules. This transformation enhances the effectiveness of original botanical drugs by improving the absorption and bioavailability of their active components in the body. As discussed above, botanical drugs are rich in chemical components, and microbial fermentation resulting in the biotransformation of botanical drugs can generate new metabolites, which involve an interaction between microorganisms and botanical metabolites that ultimately boost the activity and enhance the efficacy of the medicines.

### 3.3 Production of new metabolites

Fermentation of botanical drugs, a method of changing their composition of botanical drugs, can increase the active ingredients in MFH or produce new active metabolites through the metabolic activity of microorganisms. Probiotic bacteria can synthesize precursor compounds from the active ingredients of botanical drugs, interact with secondary metabolites of microorganisms to form new metabolites, and affect the metabolism of probiotic bacteria to produce novel metabolites. Some active ingredients are found in small amounts in plants, and the need for large quantities of valuable botanical drugs is extremely inconvenient. Direct extraction and isolation of ingredients from medicinal plants is limited by many factors, such as slow plant growth and climate change. Studies have reported that fermented botanical drugs can increase the active components of single botanical drugs, an effect that has great economic potential. Fermentation improves the pharmacological properties of botanical drugs mainly through the modification of naturally occurring molecules such as isoflavones, saponins, phytosterols, and phenols that exert beneficial health-promoting and disease-preventing effects, in keeping with the “theory of the oriental medicine.” In recent years, with the rapid progress in microbial fermentation technologies and in-depth research on the modernization of botanical drugs, microbial fermentation and the transformation of botanical drugs have gained considerable interest and have emerged as new approaches to produce novel active metabolites with potent medicinal value (Wu et al., 2013). The fermentation of medicinal botanical drugs is a decomposition process carried out using microorganisms such as bacteria and fungi. This process is regarded as a valuable biocatalytic technique for producing novel, active, and less toxic bioactive products that may be difficult to obtain using biological systems or chemical synthesis. Shakya et al. fermented *P. lactiflora* extract from *Lactobacillus shortcombii* 174A significantly increases gallic acid content. Additionally, a newly generated bioactive metabolite from fermentation was identified as pyrogallol, demonstrating its ability to inhibit inflammatory responses (Shakya et al., 2021). Many bacterial and yeast strains are used in the fermentation of botanical drugs. *Bacillus subtilis* and *S. cerevisiae* are the two most commonly used strains. The fermentation of *Panax notoginseng* with *Bacillus subtilis* produces a new active substance (ginsenoside RH4), and the fermentation of *A. membranaceus* with *Bacillus subtilis* results in a much higher polysaccharide content and an increased immune-enhancing effect. It has been proposed that bacterial fermentation not only generates bioactive metabolites of flavonoids but also alters their structure. This process includes the deglycosylation, sulfation, or methylation of flavonoids, ultimately influencing their absorption rate and metabolism in the liver (Scalbert and Williamson, 2000). Bacterial fermentation-induced alterations in flavonoid structures can enhance absorption rates and overall absorption levels. This may lead to an increased bioactivity and bioavailability of the active ingredients (Hendrich, 2002), potentially resulting in beneficial effects on bone health (Scholz-Ahrens et al., 2007). Oh et al. found that compared to unfermented WuYao ShunQi San (a traditional Botanical formula consisting of 12 botanical drugs), WuYao ShunQi San fermented by *Lactobacillus* was effective in inhibiting the production of pro-inflammatory mediators such as NO, prostaglandin E2, TNF-a and IL-6, as well as their respective synthase-inducible nitric oxide synthase and cyclooxygenase-2, thus exhibiting potent anti-inflammatory effects. This study showed that fermentation leads to the production of five pro-inflammatory mediators: Nitric Oxide Synthase and Cyclooxygenase-2. The study showed that fermentation led to an increase in the concentration of five unknown compounds, whereas the glycyrrhizin content in the fermented WuYao ShunQi San decreased dramatically to undetectable levels (Oh et al., 2012). Sheih et al. found that fermentation of *A. membranaceus* using *Aspergillus* spp. resulted in a significant increase in the phenolic content of *A. membranaceus*. Fermented *A. membranaceus* showed stronger antioxidant activity against various free radicals than unfermented *A. membranaceus*. In addition, a potent novel phenolic antioxidant, 3,4-bis(4′-hydroxyphenyl) isobutyric acid, with a molecular weight of 272, was isolated from the methanolic extract of fermented *A. membranaceus* (Sheih et al., 2011). Additionally, a notable increase in the concentration of saponin-D, flavonoids, and polyphenols was observed during the fermentation of *Platycodon grandiflorus* root using *L. rhamnosus* 217-1 (Wang et al., 2022b).

### 3.4 Reutilization of botanical residues

After the extraction of botanical drugs, leftover dregs are often disposed of by dumping, leading to environmental pollution and underutilization of the active ingredients. The increasing production of botanical drugs owing to their growing prevalence of botanical drugs globally poses a significant challenge. Improper handling not only wastes resources, but also contributes to environmental pollution. Botanical drug fermentation has emerged as a promising solution for converting these dregs into valuable resource materials, offering great potential for further development and utilization. Kong et al. selected two endophytic actinomycetes and three endophytic fungi to determine their potential ability to reuse Huazhenghuisheng oral-liquid (HOL) residues. HPLC analyses showed that all endophytic bacteria could produce metabolites from HOL residues, with the most abundant metabolites produced by *Aspergillus cristatus* CB10002. Further studies showed that *A. cristatus* CB10002 could reuse the composite HOL residue. Nine anthraquinone compounds with medicinal value were produced (Kong et al., 2019). As by-products of extraction with water or ethanol, botanical drug residues still contain approximately 30%–50% of the medicinally active substances (Meng et al., 2017). The fermentation of botanical drugs not only enhances potency and detoxification but also utilizes leftover medicinal dregs as a culture substrate. This approach not only addresses potential environmental pollution issues caused by dregs, but also maximizes the proteins and sugars present in them, thereby reducing production costs and optimizing herb resource utilization. Natural environmental factors and human intervention have decreased the production and quality of several commonly used botanical drugs. To maximize the utilization of traditional botanical drug resources, it is important to minimize resource loss. In the case of precious and endangered botanical drugs, identifying and utilizing suitable alternatives can help conserve herbal resources and protect the ecological balance, promoting the sustainable utilization of botanical drug resources.

## 4 Common strains of botanical drugs fermentation

Microbial fermentation of botanical drugs has a rich historical background and has recently gained significant attention in modernization research on fermented botanical drugs. The key to successful fermentation technology for botanical drugs lies in the careful selection and cultivation of high-quality strains. The types and quantities of microbial secondary metabolites are closely linked to the strains used. Additionally, enzyme systems or secondary metabolites produced by exotic bacterial strains can interact with the intestinal flora of the human body and other related targets, thereby regulating various body functions and providing therapeutic effects. Therefore, the selection of strains with high yields, efficient conversion, and minimal adverse reactions is crucial for the fermentation of botanical drugs. When selecting microorganisms for the fermentation of botanical drugs, the characteristics of botanical drugs, purpose of fermentation, genetic background and growth characteristics of microorganisms, and actual production conditions should first be considered comprehensively to ensure that the fermentation effect and efficacy of botanical drugs are maximized.

Currently, the microorganisms predominantly utilized in the fermentation of botanical drugs fall into two main categories: fungi and bacteria. Fungi exhibit a notable capacity to break down the active compounds present in botanical drugs under straightforward culture conditions and minimal environmental requirements. Compared with fungal fermentation, bacterial fermentation is characterized by its diversity, rich metabolite content, simple structure, environmental sensitivity, and ease of enhancement. At present, the common microorganisms are *Bacillus* species, such as *Bacillus subtilis*, which have strong resistance and vitality, are able to survive in extreme environments, and produce a variety of enzymes and active substances, which can help the conversion and extraction of botanical drug ingredients; *Lactobacillus*: lactobacilli are often used in botanical drug fermentation to improve the taste and efficacy of botanical drugs, and at the same time, can regulate the balance of the intestinal flora to improve the body’s yeasts, such as brewer’s yeast, which have a faster growth rate and higher metabolic activity, which can promote the release and transformation of active ingredients in botanical drugs. Moulds: *A. niger*, Aspergillus oryzae, and others Moulds can produce a variety of enzymes and secondary metabolites during the fermentation of botanical drugs, which can facilitate the decomposition and transformation of botanical drug ingredients and improve their efficacy.

## 5 Influencing factors in the fermentation process of botanical drugs

Botanical drugs is a complex and delicate process, and its effect is jointly influenced by many factors. Firstly, temperature is one of the key parameters in the fermentation process, most microorganisms grow best at around 37°C. However, in the actual fermentation process, the optimal temperature may vary depending on the strain of bacteria and the raw materials of the botanical drugs. Second, the fermentation time should be determined according to the specific growth characteristics of the selected strains; some strains may be more sensitive to specific fermentation times, and a short fermentation time may lead to the failure of full growth and metabolism of the strains, while an excessively long fermentation time may cause aging of the strains or the production of undesirable metabolites. Table 1 lists the effects of temperature and time on the fermentation effect during the fermentation of botanical drugs. In practice, it is necessary to optimize fermentation temperature and time through experimental exploration to obtain the best fermentation effect and product quality.

**TABLE 1:** Fermentation conditions of selected botanicals.

Botanical drug	Fermentation strains	Temperature and time	Chemical component	Pharmacological effects	References
*Coix lacryma-jobi*	*Lactobacillus plantarum* NCU137	37°C for 36 h	Free amino acids, free fatty acids ↑ 2-pentylfuran ↓	Reduced toxicity	Yin et al. (2020)
*Gynoacemma pentaphllum*	*Lactobacillus* sp. Y5	35°C for 6 days	Anthraquinones, polysaccharides, cyclic allyl glycosides ↑	Anti-diabetic	Liu et al. (2023)
*Platycodon grandiflorum*	*Lactobacillus rhamnosus* 217-1	37°C for 24 h	Polyphenols ↑ flavonoids ↑	Immune enhancement, anti-inflammatory	Wang et al. (2022b)
*Momordica charantia*	*Lactobacillus plantarum*	37°C for 48 h	Propionic acid, butyric acid, acetic acid, short-chain fatty acids ↑	Anti-diabetic	Gao et al. (2019)
*Astragalus membranaceus*	*Lactobacillus plantarum*	36°C for 36 h	Raffinose, progesterone, uridine ↑	Regulation of the intestinal flora	Li et al. (2022)
*Panax ginseng*	*Lactobacillus brevis*	37°C for 2 days	Uronic acids, polyphenols, flavonoids ↑	Anti-oxidant	Lee et al. (2012)
*Astragalus membranaceus*	*Lactobacillus plantarum*	37°C for 15 h	Nitric oxide, hydrogen peroxide ↓	Anti-inflammatory	Park et al. (2021)
*Panax ginseng*	*Lacto. plantarum* KP-4	37°C for 18 days	Ginsenosides Rg3, F2, Rh1 ↑	Anti-inflammatory	Fan et al. (2021)
*Paeonia lactiflora*	*Lactobacillus shortus* 174A	37°C for 48 h	Total phenolic content, gallic acid content ↑	Anti-inflammatory	Shakya et al. (2021)
*Morus alba*	*Lactobacillus acidophilus* A4	37°C for 18 h	Mucin production ↑ myeloperoxidase levels ↓	Anti-inflammatory	Oh et al. (2017)
*Coptis chinensis*	*Lactobacillus mesenteroides*	35.4°C for 24 h	Endotoxin levels ↓	Anti-inflammatory	Bose et al. (2012)
*Panax ginseng*	*Lactobacillus*	40°C for 12 h	Ginsenoside Rh2 content ↑	Anti-diabetic	Kim et al. (2011a)
GeGen QinLian Tang	*Saccharomyces cerevisiae*	28°C for 48 h	Flavone aglycones, puerarin ↑	Anti-diabetic	Yan et al. (2018)
DangGui BuXue Tang	*Lactobacillus plantarum*	37°C for 24 h	Α-glucosidase ↓	Anti-diabetic	Guo et al. (2020)
WuYao ShunQi San	*Lactobacillus*	37°C for 48 h	—	Anti-inflammatory	Oh et al. (2012)

## 6 Limitations and risks of fermented botanical drugs

While exploring the promising applications of fermented botanical drugs, it is important not to overlook their inherent limitations and potential risks, as this is crucial to ensure the efficiency of the fermentation process and the stability of the product quality and safety.

### 6.1 Limitations of fermented botanical drugs

The fermentation of botanical drugs may be limited by the limited resources of strains for use in the fermentation process. Moreover, the stability and activity of the microorganisms are susceptible to environmental parameters, such as temperature, humidity, and pH value, which directly lead to uncertainty about the fermentation effect and the fluctuation of the quality of the product. Although modern fermentation technology has been able to realize a certain degree of parameter monitoring and regulation, it is still a difficult task to completely eliminate the uncertainty in the fermentation process, because it is difficult to accurately control the parameters in the fermentation process, which may affect the quality and stability of the final fermentation products. In addition, the complexity of the composition of botanical drugs seriously affects the quality of the fermented products. Not all components can be effectively utilized or transformed during the fermentation process, and some of the active ingredients may be lost due to inappropriate fermentation conditions. Meanwhile, the newly produced components may have unknown pharmacological effects or safety issues. Currently, the fermented botanical drug industry also faces problems of low process standardization and the lack of unified quality standards and testing methods, which directly increases the difficulty with product quality control. The fermentation process also requires a long period of time and incurs a high cost, due to strain cultivation and fermentation equipment and raw material procurement. In addition, energy consumption during the fermentation process and waste disposal are issues that need to be considered.

### 6.2 Risks of fermented botanicals

The fermentation of botanical drugs carries risks that should not be overlooked. Some botanical drugs themselves contain toxic components, and the fermentation process may produce new toxic components or increase the levels of the original toxic components, which may pose a potential threat to human health and require rigorous safety assessments. In addition, new components produced during the fermentation process may trigger allergic reactions in humans; therefore, adequate allergy tests and risk assessments are required before their use. Meanwhile, fermented botanical drugs may interact with other drugs, affecting their efficacy or increasing the risk of adverse reactions. Therefore, special attention needs to be paid to drug compounding contraindications when combining drugs. Owing to differences between individual patients and different disease states, there may be uncertainty in the efficacy of fermented botanical drugs. Therefore, adequate efficacy assessments and monitoring are required for clinical applications.

## 7 Conclusion and prospects

With continuous and in-depth study of botanical drug fermentation, this technology is expected to add value to botanical drugs as a novel approach. Through fermentation, the active ingredients of botanical drugs can be transformed and concentrated, the content of active substances increases, and new active ingredients can be produced, thus broadening the clinical application of botanical drugs. For example, polysaccharides and saponins in certain botanical drugs that have undergone fermentation, such as *A. membranaceus* and *A. sinensis*, which are fermented using specific strains of microorganisms, are converted into new substances with greater anti-tumor activity. These new substances can inhibit tumor cell growth. The components of botanical drugs obtained through fermentation techniques, such as certain flavonoids, have shown powerful effects in the inhibition of inflammatory responses.

The potential toxicity of medicines can be reduced, making botanical drugs safer and more effective, and the amount of medicinal materials can be reduced, enabling the multi-level use of botanical drugs. Fermentation technology can transform and modify complex chemical components in botanical drugs through the metabolic activities of microorganisms and act on the toxic components of botanical drugs to change their structure, thus reducing their toxicity. Simultaneously, the interactions between microorganisms and certain components of traditional botanical drugs may produce new substances with improved pharmacological effects and lower toxicity. Fermentation technology can also improve the taste and stability of botanical drugs to increase their safety and allow them to be easily accepted by patients and used for a long time.

In addition, the fermentation of traditional botanical drugs can also expand the scope of application of traditional botanical drugs, so that they can play a greater role in the fields of beauty, healthcare, etc., Botanical drugs are rich in active ingredients that are beneficial to the skin such as polyphenols and flavonoids, which are often found in plant forms and are unfavorable for direct skin absorption. Through fermentation, microbial metabolism can convert these active ingredients into forms that are more easily absorbed by the skin. For example, the antioxidant capacity of certain botanical drugs is significantly enhanced after fermentation, which can effectively resist damage caused by free radicals to the skin and slow skin aging. Fermented botanical drugs can also improve the intestinal microecological environment, promote the value-added of beneficial bacteria, and improve intestinal levels, thereby enhancing overall immunity.

It is important to note that most of the current research on fermented botanical drugs remains focused on simple pharmacological function demonstration, and the exact mechanism of the health benefits of these fermentation products has not yet been elucidated; therefore, there is still a lack of systematic mechanism research and clinical trials. Most studies on fermented botanical drugs have limitations, such as insufficient standardization of experimental design, backwardness of fermentation process detection, control, and technology, unknown mechanism of interaction between microorganisms and botanical drugs, imperfect evaluation system of pharmacological efficacy, and uneven quality of the literature. To promote in-depth research on the fermentation of botanical drugs, it is necessary to improve and innovate against these limitations in the future.

To summarize, the research progress of botanical drug fermentation technology not only enriches botanical drug preparation but also provides strong support for the improvement of the pharmacological effects of botanical drugs and the expansion of their applications. In the future, as botanical drug fermentation technology will continue to improve and innovate, its role in the modernization and internationalization of botanical drugs will be more prominent. For example, genomics, transcriptomics, metabolomics, proteomics, and other multi-omics analysis techniques can be used to study the biological mechanisms involved in the fermentation process of traditional botanical drugs and provide a scientific basis for optimizing the fermentation technology. The rapid development of intelligent and automated technology has brought new prospects for research on botanical drug fermentation. Through the integration of artificial intelligence, machine learning, and other technologies, the fermentation process of botanical drugs can be accurately regulated, fermentation conditions improved, and fermentation efficiency enhanced. Fermentation technology can also be used to produce novel botanical drugs with unique medicinal and biological properties. Fermentation technology can be used to develop functional foods and botanical drug health products by combining yeast and probiotics, which are beneficial to health. By improving the efficacy and reducing the toxicity of botanical drugs through fermentation technology, botanical drugs can become more competitive in the global pharmaceutical market and provide safer and more effective treatment options for patients worldwide, thereby making a greater contribution to human health. All botanical drugs name and family were validated using http://www.plantsoftheworldonline.org and Chinese Pharmacopoeia as shown in Table 2.

**TABLE 2:** Botanical names and medicinal parts.

Scientific name of the drug	Family	Medicinal part	References
*Aloe barbadensis* Miller	Asphodelaceae	Herba	Chinese Pharmacopoeia Commission. (2020)
*Ampelopsis japonica* (Thunb.) Makino	Vitaceae	Radix	Chinese Pharmacopoeia Commission. (2020)
*Anemarrhena asphodeloides* Bge	Asparagaceae	Rhizoma	Chinese Pharmacopoeia Commission. (2020)
*Angelica sinensis* (Oliv.) Diels	Apiaceae	Radix	Chinese Pharmacopoeia Commission. (2020)
*Arctium lappa* L	Asteraceae	Fructus	Chinese Pharmacopoeia Commission. (2020)
*Aristolochia debilis* Sieb.et Zucc	Aristolochiaceae	Fructus	Chinese Pharmacopoeia Commission. (2020)
*Astragalus membranaceus* (Fisch.) Bge	Fabaceae	Radix	Chinese Pharmacopoeia Commission. (2020)
*Atractylodes macrocephala* Koidz	Asteraceae	Rhizoma	Chinese Pharmacopoeia Commission. (2020)
*Bletilla striata* (Thunb.) Reichb.f	Orchidaceae	Rhizoma	Chinese Pharmacopoeia Commission. (2020)
*Broussonetia papyrifera* (L.) Vent	Moraceae	Fructus	Chinese Pharmacopoeia Commission. (2020)
*Citrus aurantium* L	Rutaceae	Fructus	Chinese Pharmacopoeia Commission. (2020)
*Coix lacryma-jobi* L.var.ma-yuen (Roman.) Stapf	Poaceae	Semen	Chinese Pharmacopoeia Commission. (2020)
*Coptis chinensis* Franch	Ranunculaceae	Rhizoma	Chinese Pharmacopoeia Commission. (2020)
*Curcuma longa* L	Zingiberaceae	Rhizoma	Chinese Pharmacopoeia Commission. (2020)
*Dictamnus dasycarpus* Turcz	Rutaceae	Cortex	Chinese Pharmacopoeia Commission. (2020)
*Dimocarpus longan* Lour	Sapindaceae	Fructus	Chinese Pharmacopoeia Commission. (2020)
*Ganoderma lucidum* (Leyss.ex Fr.) Karst	—	Fructus	Chinese Pharmacopoeia Commission. (2020)
*Ginkgo biloba* L	Ginkgoaceae	Folium	Chinese Pharmacopoeia Commission. (2020)
*Glycine max* (L.) Merr	Fabaceae	Semen	Chinese Pharmacopoeia Commission. (2020)
*Glycyrrhiza glabra* L	Fabaceae	Radix et rhizoma	Chinese Pharmacopoeia Commission. (2020)
*Gynoacemma pentaphllum* (Thunb) Mak	Rubiaceae	Herba	Chinese Pharmacopoeia Commission. (2020)
*Hippophae rhamnoides* L	Elaeagnaceae	Fructus	Chinese Pharmacopoeia Commission. (2020)
*Huperzia serrata* (Thunb.) Trevis	Lycopodiaceae	Cortex	Kozikowski and Tückmantel. (1999)
*Laminaria japonica* Aresch	—	Thallus	Chinese Pharmacopoeia Commission. (2020)
*Lycium barbarum* L	Solanaceae	Fructus	Chinese Pharmacopoeia Commission. (2020)
*Lepidium meyenii* Walp	Brassicaceae	Rhizoma	Toledo (1998)
*Magnolia officinalis* Rehd.et Wils	Magnoliaceae	Cortex	Chinese Pharmacopoeia Commission. (2020)
*Momordica charantia* L	Cucurbitaceae	Fructus	Raman and Lau. (1996)
*Morus alba* L	Moraceae	Ramulus	Chinese Pharmacopoeia Commission. (2020)
*Nelumbo nucifera* Gaertn	Nelumbonaceae	Semen	Chinese Pharmacopoeia Commission. (2020)
*Paeonia lactiflora* Pall	Paeoniaceae	Radix	Chinese Pharmacopoeia Commission. (2020)
*Panax ginseng* C.A.Mey	Araliaceae	Radix et rhizoma	Chinese Pharmacopoeia Commission. (2020)
*Panax notoginseng* (Burk.) F.H.Chen	Araliaceae	Radix et rhizoma	Chinese Pharmacopoeia Commission. (2020)
*Perilla frutescens* (L.) Britt	Lamiaceae	Fructus	Chinese Pharmacopoeia Commission. (2020)
*Platycodon grandiflorum* (Jacq.) ADC.	Campanulaceae	Radix	Chinese Pharmacopoeia Commission. (2020)
*Polygonatum sibiricum* Red	Asparagaceae	Rhizoma	Chinese Pharmacopoeia Commission. (2020)
*Poria cocos* (Schw.) Wolf	—	Sclerotium	Chinese Pharmacopoeia Commission. (2020)
*Portulaca oleracea* L	Portulacaceae	Herba	Chinese Pharmacopoeia Commission. (2020)
*Pueraria lobata* (Willd.) Ohwi	Fabaceae	Radix	Chinese Pharmacopoeia Commission. (2020)
*Rosa roxburghii* Tratt	Rosaceae	Flos	Wang et al. (2021)
*Salvia miltiorrhiza* Bge	Lamiaceae	Rhizome	Chinese Pharmacopoeia Commission. (2020)
*Taraxacum mogolicum* Hand.-Mazz	Asteraceae	Herba	Chinese Pharmacopoeia Commission. (2020)
*Tribulus terrestris* L	Zygophyllaceae	Fructus	Chinese Pharmacopoeia Commission. (2020)
*Zingiber officinale* Rosc	Zingiberaceae	Rhizome	Chinese Pharmacopoeia Commission. (2020)
*Ziziphus jujuba* Mill.var.spinosa (Bunge) Hu ex H.F.Chou	Rhamnaceae	Semen	Chinese Pharmacopoeia Commission. (2020)
